# Rehabilitation improves the effectiveness of nusinersen in children with type 2 spinal muscular atrophy: pNF-H and muscle MRI as potential biomarkers

**DOI:** 10.3389/fneur.2025.1549587

**Published:** 2025-04-11

**Authors:** Yifan Sun, Wei Li, Xin Cui, Yang Li, Xiucheng Gao, Dalin Fu, Xiaoke Zhao, Tong Cao, Min Zhu

**Affiliations:** ^1^Department of Rehabilitation, Children's Hospital of Nanjing Medical University, Nanjing, Jiangsu, China; ^2^Department of Neurology, Children's Hospital of Nanjing Medical University, Nanjing, Jiangsu, China; ^3^Department of Radiology, Children's Hospital of Nanjing Medical University, Nanjing, Jiangsu, China; ^4^Department of Clinical Laboratory, Children's Hospital of Nanjing Medical University, Nanjing, Jiangsu, China

**Keywords:** type-2 spinal muscular atrophy, muscle magnetic resonance imaging, nusinersen, rehabilitation therapy, phosphorylated neurofilament heavy chain

## Abstract

**Introduction:**

Rehabilitation therapy is an important approach for spinal muscular atrophy (SMA) management. Currently, rare articles introduce that the combination of nusinersen and rehabilitation yields better results in SMA patients compared to using nusinersen alone. The present study examined whether rehabilitation therapy can improve the effectiveness of nusinersen and phosphorylated neurofilament heavy chain (pNF-H) and muscle magnetic resonance imaging (MRI) can serve as potential biomarkers for evaluating the therapeutic effects in type-2 SMA patients.

**Methods:**

This observational study enrolled 22 pediatric patients with type-2 SMA. Enrolled patients were divided into two groups based on the rehabilitation treatment. Motor function and swallowing function were analyzed at baseline, 6, 10, and 14 months. The level of pNF-H and MRI of the thigh skeletal muscles were analyzed at baseline and 14 months.

**Results:**

Greater improvement in motor function was observed in the rehabilitation group compared with the non-rehabilitation group. The levels of pNF-H in the serum and cerebrospinal fluid significantly decreased at 14 months. One patient from the rehabilitation treatment group showed mild improvement in the degree of fatty infiltration in the quadriceps muscles after 14 months.

**Conclusion:**

This study suggests that rehabilitation therapy improves the effectiveness of nusinersen on type-2 SMA patients, and the levels of pNF-H and skeletal muscle MRI can serve as potential biomarkers for evaluating the effectiveness of SMA treatment.

## 1 Introduction

Spinal muscular atrophy (SMA) is a rare autosomal recessive neuromuscular disorder caused by homozygous absence of the survival motor neuron 1 (SMN1) gene (1). Alterations in the SMN1 gene result in insufficient production of functional SMN proteins, leading to degeneration of spinal motor neurons in the anterior horn and subsequent muscle weakness and atrophy (1). Although the SMN2 gene is homologous to the SMN1 gene in the human body, approximately 90% of transcripts lack exon 7 due to alternative splicing of the SMN2 gene, which produces SMN proteins that are non-functional and rapidly degraded (2).

Nusinersen is the first approved disease-modifying drug for the treatment of SMA. It is a modified antisense oligonucleotide that targets the intronic splicing silencer N1 site in the SMN2 intron 7 downstream of exon 7 in the precursor mRNA and competes with the binding of heterogeneous nuclear ribonucleoprotein A1 (3–5). This enhances the inclusion of exon 7 in the transcripts, thereby increasing the production of functional SMN protein (6). Clinical trials of nusinersen have provided evidence on its effectiveness in terms of motor function improvements as well as good tolerability (7–9).

Rehabilitation therapy is an important component of care for patients with SMA. Common rehabilitation modalities include physical therapy, use of orthotic devices, and occupational therapy (10). Targeted training can prevent and delay secondary symptoms such as joint contractures, spinal deformities, and mobility impairments, thereby improving the quality of life for affected children. Previous studies have shown that rehabilitation therapy improves the motor function of patients with SMA before the market launch of disease-modifying drugs (11, 12). However, rehabilitation training is not able to reverse the progression of the disease. Currently, rare article introduces that the combination of medication and rehabilitation yields better results in SMA patients compared to using medication alone (13).

The rise of treatment options led to a concomitant need of biomarkers for therapeutic guidance and an improved disease monitoring. The most promising markers include appliance-based measures such as electrophysiological and imaging-based indices as well as molecular markers including SMN-related proteins and markers of neurodegeneration and skeletal muscle integrity (14). Phosphorylated neurofilament heavy chain (pNF-H) is a biomarker that indicates axonal degeneration in neurological disorders (15). Increased levels of pNF-H in the cerebrospinal fluid (CSF) and plasma/serum have been observed in patients with motor neuron diseases. Previous studies have supported the use of pNF-H in plasma/serum and cerebrospinal fluid as a potential biomarker for evaluating the efficacy of nusinersen in patients with SMA (16, 17).

Muscle magnetic resonance imaging (MRI) is an effective non-invasive tool for assessing the extent of muscle involvement in neuromuscular diseases. MRI examination of the thigh skeletal muscles in SMA patients can further track muscle atrophy and fatty infiltration (18).

We evaluated the effectiveness and safety of nusinersen combined with rehabilitation therapy in a group of Chinese patients with SMA type-2 in a real-world clinical setting, and explored the reliability of pNF-H and muscle MRI as biomarkers of SMA progression.

## 2 Materials and methods

### 2.1 Study design and participants

This observational study was conducted at the Children’s Hospital of Nanjing in China. All patients were genetically confirmed to have SMA, and are classified as type 2 SMA based on clinical presentation (onset before 18 months of age, ability to sit independently, but inability to stand or walk) (19). These patients aged 1–14 years and had a minimum treatment duration of 14 months. Patients with severe comorbidities (such as severe liver, kidney, heart, or lung failure, and severe coagulation disorders) and those who were unable to cooperate with study procedures were excluded. Enrolled patients were divided into two groups based on the rehabilitation treatment: 11 patients who received systematic rehabilitation treatment were assigned to the rehabilitation group, and 11 patients who did not receive systematic rehabilitation therapy were included in the non-rehabilitation group. Among these 22 patients, 10 had 3 copies of the SMN2 gene, while the SMN2 copy number was unknown for the remaining patients. Systematic rehabilitation training includes upper and lower limb physical therapy (PT), occupational therapy (OT), respiratory training, orthotic device wearing, standing training, intensive suspension weight reduction training, and lower limb rehabilitation cycling. PT includes exercise training (based on the child’s muscle strength, appropriate assistance can be provided through active, anti-gravity, or progressive resistance training), joint stretching training, and balance training. Respiratory training includes respiratory muscle training, maintaining thoracic compliance training, and coughing and sputum clearance training. Each session lasts 30 min, at least five times per week. The study was approved by the Ethics Committee of the Children’s Hospital of Nanjing (approval number: 202307004-1). All participants provided written informed consent.

### 2.2 Experimental procedure

Nusinersen was administered by lumbar puncture with intrathecal injection. A 5-mL solution containing 12 mg of nusinersen was injected into the intrathecal space. Four loading doses were given one each on day 0, day 14, day 28, and day 63, followed by maintenance doses every 4 months (6). Baseline demographic and clinical characteristics, including age, gender, SMN2 gene copy number, and baseline motor function scales were collected. The data on motor function at baseline and at 6, 10, and 14 months of nusinersen treatment were also collected. Adverse events were recorded throughout the study. The data on swallowing function at baseline and at 14 months were collected.

### 2.3 Clinical assessments

Effectiveness clinical assessments included motor function and swallowing function was evaluated using the Children’s Hospital of Philadelphia Infant Test of Neuromuscular Disorders (CHOP-INTEND) (maximum score of 64 points) (20), Hammersmith Functional Motor Scale-Expanded (HFMSE) (maximum score of 66 points) (21), Revised Upper Limb Module (RULM) (maximum score of 37 points) (22), and Hammersmith Infant Neurological Examination-2 (HINE-2) (maximum score of 26 points) (23) scores. Swallowing function was assessed using the Fujishima Dysphagia Scale (FDS) (maximum score of 10 points) (24). The selected motor function scales (CHOP-INTEND, HFMSE, RULM, HINE-2) have been validated for use in pediatric SMA populations across the studied age range (1–14 years) (25). CHOP-INTEND is specifically designed for infants and non-ambulatory children (20), while HFMSE and RULM have demonstrated reliability in older children with SMA type 2 (21, 22). HINE-2 was applied with age-appropriate adaptations as per standardized protocols (23).

Safety assessments included central nervous system reactions (headache, drowsiness, and insomnia), gastrointestinal reactions (vomiting, abdominal pain, and diarrhea), allergic reactions (rash and swelling), back pain, joint pain, electrocardiogram abnormalities, liver and renal function abnormalities, and coagulation function abnormalities.

### 2.4 Collection and analysis of pNF-H in the serum and cerebrospinal fluid

The levels of pNF-H in the serum and cerebrospinal fluid were assessed. Serum and cerebrospinal fluid samples were collected at baseline and 14 months, and the samples were frozen at −80°C. The concentration of pNF-H was measured using a commercially available, enzyme-linked immunosorbent assay (ELISA) kit (JSBOSSEN), following the manufacturer’s instructions.

### 2.5 MRI data acquisition

The MRI examination of the thigh skeletal muscles was also conducted. Muscle MRI examinations of thighs were performed at 1.5/3.0 T. T1-weighted MR images were used to evaluate muscle involvement. The degree of muscle fat infiltration is semi-quantitatively evaluated according to the modified Mercuri grading system (26). Specifically, the grading of muscle fat infiltration is as follows: Grade 0, no abnormal high signal lesions within the skeletal muscles; Grade 1, scattered small high signal lesions within the skeletal muscles; Grade 2, scattered high signal lesions within the skeletal muscles, with affected lesions accounting for less than 30% of muscle volume; Grade 3, scattered confluent high signal lesions within the skeletal muscles, with affected lesions accounting for 30% to 60% of muscle volume; Grade 4, large confluent high signal lesions within the skeletal muscles, with affected lesions exceeding 60% of muscle volume, but with remaining muscle tissue present in the surrounding area; Grade 5, diffuse high signal lesions throughout the skeletal muscles, with muscles completely replaced by connective tissue and fat.

### 2.6 Statistical analysis

A statistical analysis was performed using the SPSS software. Normal distribution data is represented by mean [standard deviation (SD)], while non-normal distribution data is represented by median (P25,P75). The Wilcoxon signed-rank test and Paired Samples t-test were used to compare the changes in effectiveness outcomes before and after treatment. The Mann–Whitney U test and Independent Samples t-test were used to compare the differences in effectiveness outcomes between the rehabilitation group and the non-rehabilitation group. Spearman’s correlation and Pearson’s correlation were used to assess correlations between variables. All statistical tests were two-tailed, and a *p* value of less than 0.05 was considered statistically significant.

## 3 Results

### 3.1 Demographic and clinical characteristics of SMA patients

**Figure 1:**
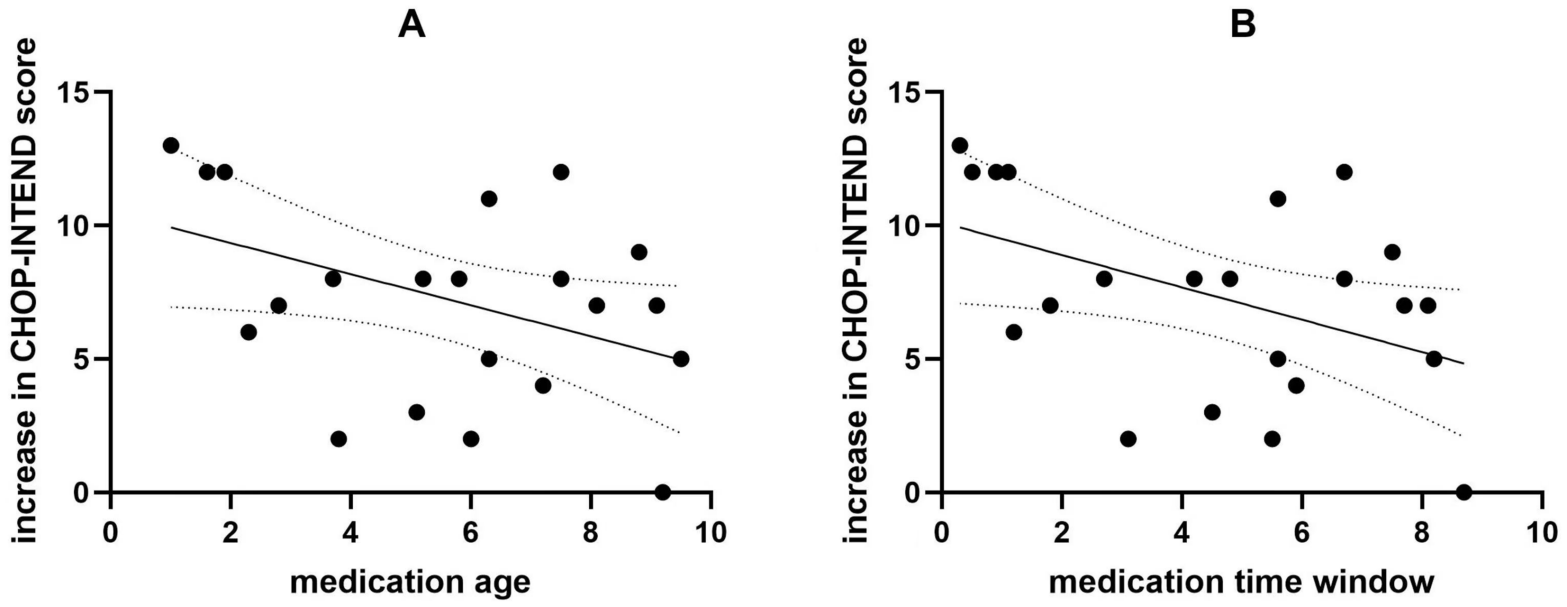
The correlation between medication age, medication time window, and increase in CHOP-INTEND scores. The medication age is negatively correlated with the increase in CHOP-INTEND scores **(A)**. The medication time window is negatively correlated with the increase in CHOP-INTEND scores **(B)**.

Between August 2021 and August 2023, 22 children with SMA type 2 who received nusinersen treatment at least 7 times and had available clinical data at baseline and 14 months were screened. Among them, 11 patients received systematic rehabilitation treatment in addition to nusinersen and 11 did not. The baseline demographic and clinical data are presented in Table 1. No significant differences were observed at baseline between the rehabilitation group and the non-rehabilitation group. We analyzed the correlation between medication age, medication time window (time from onset to medication), and increase in CHOP-INTEND scores. The results are presented in Figure 1. A negative correlation was observed between the patients’ age at medication and the increase in CHOP-INTEND score (r = −0.430, *p* = 0.046). Similarly, a negative correlation was found between the patients’ medication time window and the increase in CHOP-INTEND score (r = −0.450, *p* = 0.036).

**Table 1:** Demographic and clinical characteristics at baseline.

Characteristic	Rehabilitation group	Non-rehabilitation group	*p*-value
Age of nusinersen treatment initiation—yr, median (P25,P75)	5.2 (1.9–7.5)	6.3 (3.8–9.1)	0.217
Gender (*N*)	4 M/7F	7 M/4F	0.395
Duration of medication window—yr, median (P25,P75)	4.2 (1.1–6.7)	5.6 (3.1–8.1)	0.258
CHOP-INTEND score, median (P25,P75)	37.0 (32.0–46.0)	41.0 (32.0–52.0)	0.347
HINE-2 score Median (P25,P75)	8.0 (7.0–10.0)	11.0 (7.0–14.0)	0.276

### 3.2 Changes of swallowing function in SMA patients

Except 3 patients who had a FDS score of 8 and 2 patients who had a FDS score of 9, the remaining patients had a score of 10 on the FDS at baseline. 2 patients had a FDS score of 8 at baseline, which increased to 9 and 10, respectively, after 14 months of treatment. The FDS scores remained stable in the remaining patients.

### 3.3 Comparison of motor function between rehabilitation treatment group and non-rehabilitation treatment group in SMA patients

**Figure 2:**
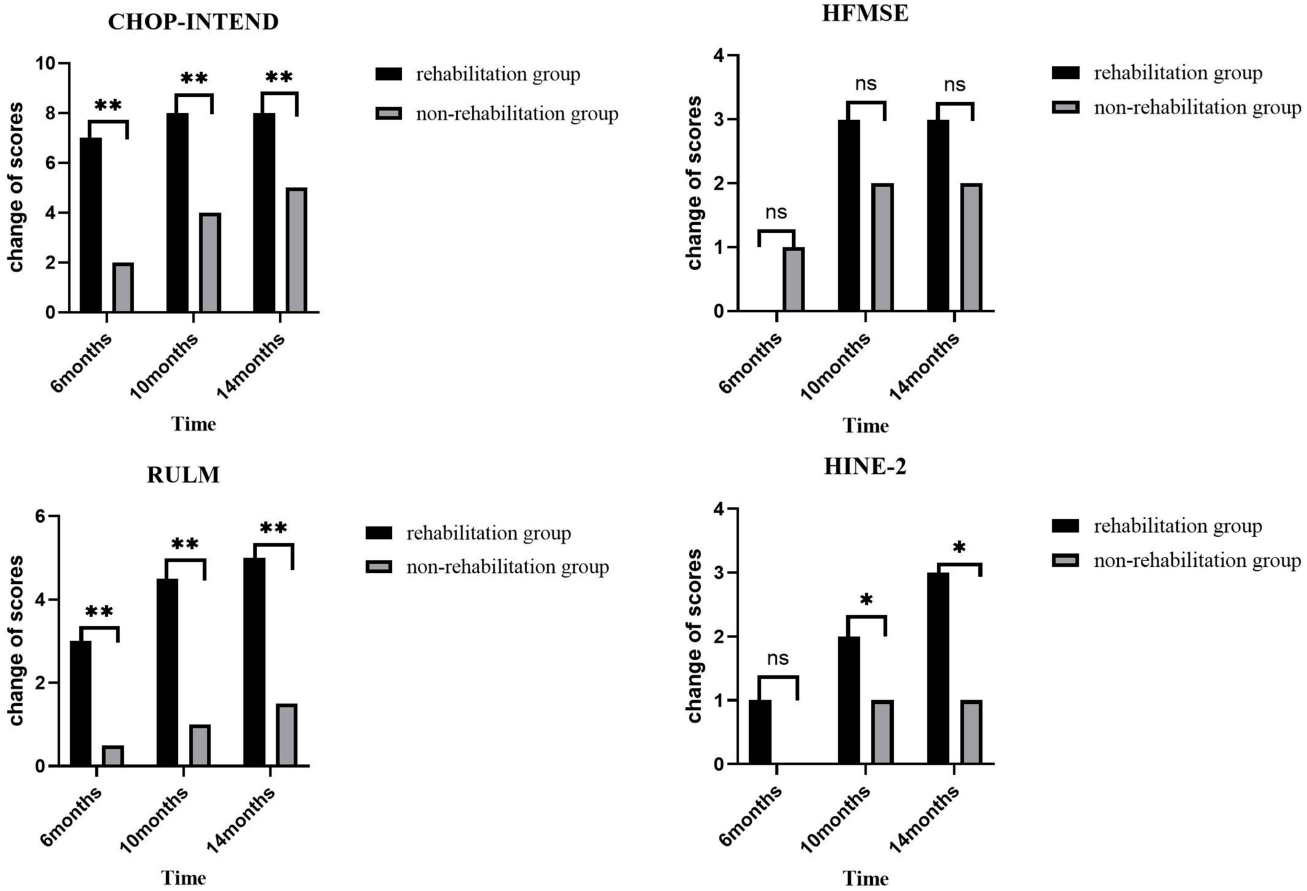
The changes from baseline in scores on various motor function scales in both groups of patients at each time point. Greater improvements in the CHOP-INTEND and RULM score at 6, 10, and 14 months from baseline were achieved by the rehabilitation group than the non-rehabilitation group. *p < 0.05 versus the non-rehabilitation group; **p < 0.01 versus the non-rehabilitation group.

The scores of motor function scales at 6, 10, and 14 months of nusinersen treatment in the rehabilitation group and the non-rehabilitation group are presented in Table 2. In the rehabilitation treatment group, the scores on various motor function scales at 6, 10, and 14 months showed significant improvement compared to baseline (*p* < 0.05). In the non-rehabilitation group, the scores of CHOP-INTEND, HFMSE, RULM showed significant improvement compared to baseline (*p* < 0.05). The scores of HINE-2 only showed significant improvement at 14 months of treatment. Changes in motor function over time in the rehabilitation group and the non-rehabilitation group are presented in Table 3 and Figure 2. Greater improvements in the CHOP-INTEND score at 6, 10, and 14 months from baseline were achieved by the rehabilitation group than the non-rehabilitation group (*p* = 0.032, *p* = 0.012, and *p* = 0.004, respectively). Similarly, the RULM scores were improved by a greater degree in the rehabilitation group than the non-rehabilitation group at the three time points after treatment (*p* = 0.002, *p* = 0.007, and *p* = 0.009, respectively). A trend of greater improvements in HFMSE and HINE-2 scores in the rehabilitation group compared with the non-rehabilitation group was observed at each observation time point after treatment, but the difference was only significant for HINE-2 at 10 months and 14 minths (*p* = 0.012 and *p* = 0.029).

**Table 2:** The scores of motor function scales at baseline, 6, 10, and 14 months of nusinersen treatment in the rehabilitation group and the non-rehabilitation group.

Characteristics	Non-rehabilitation group (*n* = 11)				Rehabilitation group (*n* = 11)			
	Baseline	6 months	10 months	14 months	Baseline	6 months	10 months	14 months
CHOP-INTEND	43.09 (10.21)	45.73 (10.49)^*^	47.45 (11.77)^*^	48.27 (12.24)^*^	39.18 (8.75)	45.00 (8.45)^*^	47.55 (8.59)^*^	48.64 (8.38)^*^
HFMSE	13.11 (8.92)	13.89 (9.27)^*^	15.22 (9.22)^*^	16.22 (9.13)^*^	11.44 (11.70)	12.33 (12.29)^*^	14.67 (11.72)^*^	14.78 (11.64)^*^
RULM	19.00 (9.68)	19.63 (9.84)^*^	20.63 (3.45)^*^	21.63 (9.75)^*^	12.00 (6.07)	15.25 (5.55)^*^	16.75 (7.26)^*^	17.63 (6.91)^*^
HINE-2	11.27 (5.31)	11.55 (5.15)	11.82 (5.58)	12.36 (6.12)^*^	9.64 (5.26)	10.82 (5.12)^*^	12.55 (5.11)^*^	13.09 (4.97)^*^

*p < 0.05 versus the baseline.

**Table 3:** Changes in scores of motor function scales from baseline in the rehabilitation group and the non-rehabilitation group at each time point.

Characteristics		Non-rehabilitation group				Rehabilitation group		
	*n*	6 months	10 months	14 months	*n*	6 months	10 months	14 months
CHOP-INTEND	11	2.00 (1.00–4.00)	4.00 (2.00–8.00)	5.00 (2.00–8.00)	11	7.00 (2.00–9.00)^#^	8.00 (5.00–11.00)^#^	8.00 (7.00–12.00)^#^
HFMSE	11	1.00 (0–1.50)	2.00 (0.50–2.50)	2.00 (1.00–4.00)	9	0 (0–2.00)	3.00 (0.50–5.50)	3.00 (1.00–5.50)
RULM	10	0.50 (0–1.00)	1.00 (0.25–2.75)	1.50 (1.00–5.00)	8	3.00 (1.25–5.50)^#^	4.50 (2.25–6.75)^#^	5.00 (4.00–8.25)^#^
HINE-2	11	0 (0–1.00)	1.00 (0–1.00)	1.00 (0–2.00)	11	1.00 (0–3.00)	2.00 (1–4.00)^#^	3.00 (1–6.00)^#^

^#p < 0.05 versus the non-rehabilitation group.^

### 3.4 Changes of pNF-H levels in SMA patients

**Figure 3:**
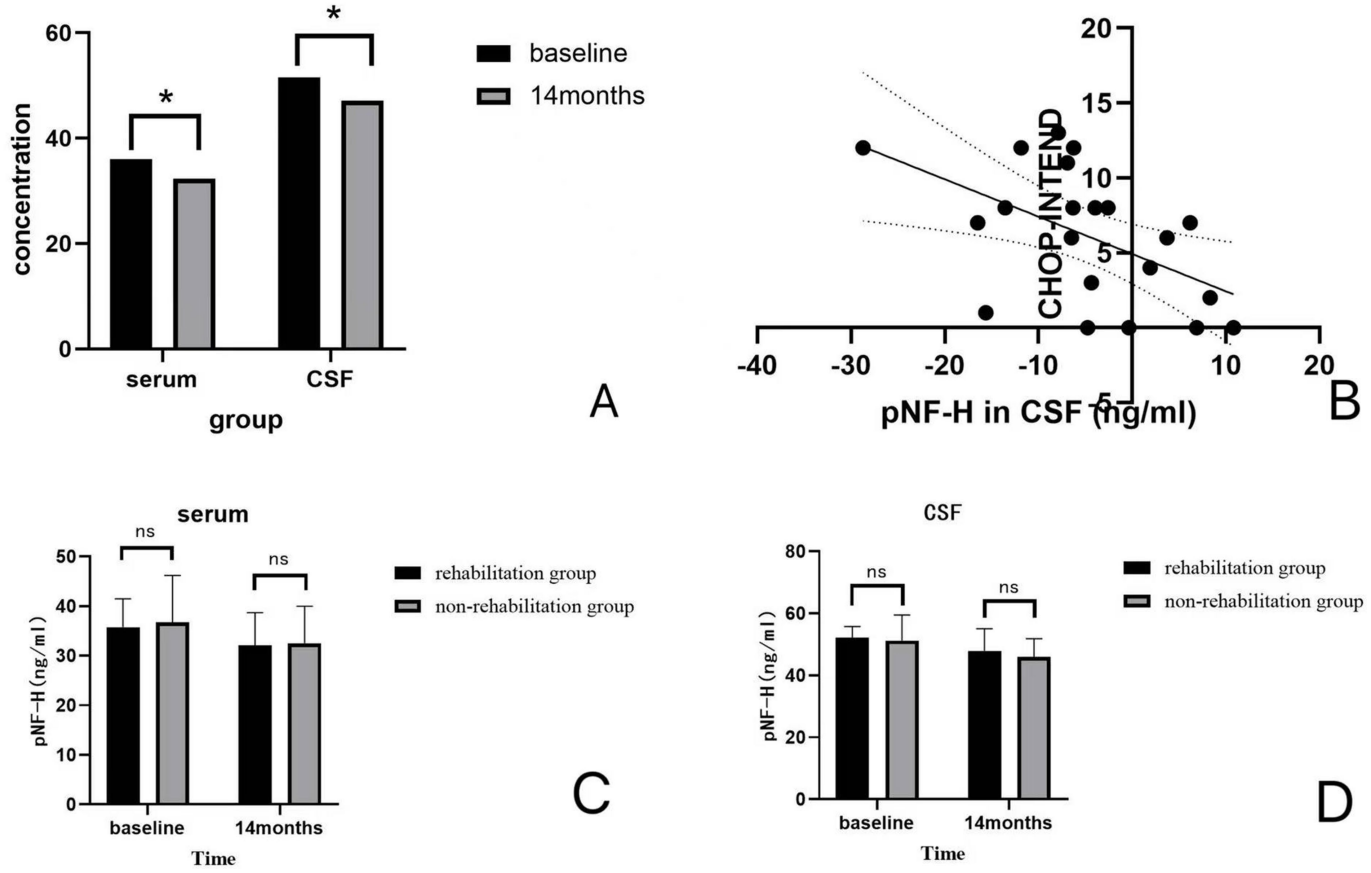
**(A)** Concentrations (ng/ml) of pNF-H in serum and CSF. A decrease in pNF-H in both serum and CSF was detected at 14 months of treatment compared with baseline. **(B)** The correlation between the change in pNF-H concentration in the CSF and increase in CHOP-INTEND scores. There is a negative correlation between the pNF-H concentration in the CSF and increase in CHOP-INTEND scores (r = −0.528, *p* = 0.014). **(C,D)** The changes in centrations of pNF-H of the patients between the rehabilitation group and the non-rehabilitation group of patients. There were no significant differences between the concentrations of pNF-H in the rehabilitation group and the non-rehabilitation group (*p* = 0.898, *p* = 0.828).

pNF-H levels in the serum and CSF samples are presented in Figure 3A. Figure 3A illustrates the concentrations of pNF-H in serum and CSF samples of the patients at baseline and at 14 months of treatment. A decrease in pNF-H in both serum and CSF was detected at 14 months of treatment compared with baseline. The concentrations of pNF-H in the serum decreased by a mean (SD) of 3.64 (0.54) ng/mL compared with baseline (*p* = 0.033), and that in the cerebrospinal fluid decreased by a mean (SD) of 4.40 (0.51) ng/mL compared with baseline (*p* = 0.046). The change in pNF-H concentration in the cerebrospinal fluid is negatively correlated with the improvement of CHOP-INTEND scores after 14 months of nusinersen treatment (r = −0.528, *p* = 0.014). The results are presented in Figure 3B. In serum, there is no correlation between the two (r = −0.082, *p* = 0.724). Figures 3C,D. compares the changes in concentrations of pNF-H of the patients between the rehabilitation group and the non-rehabilitation group of patients. There were no significant differences in the changes of pNF-H levels between the two groups (*p* = 0.898, *p* = 0.828).

### 3.5 Modified Mercuri grading of quadriceps femoris MRI in SMA patients

**Figure 4:**
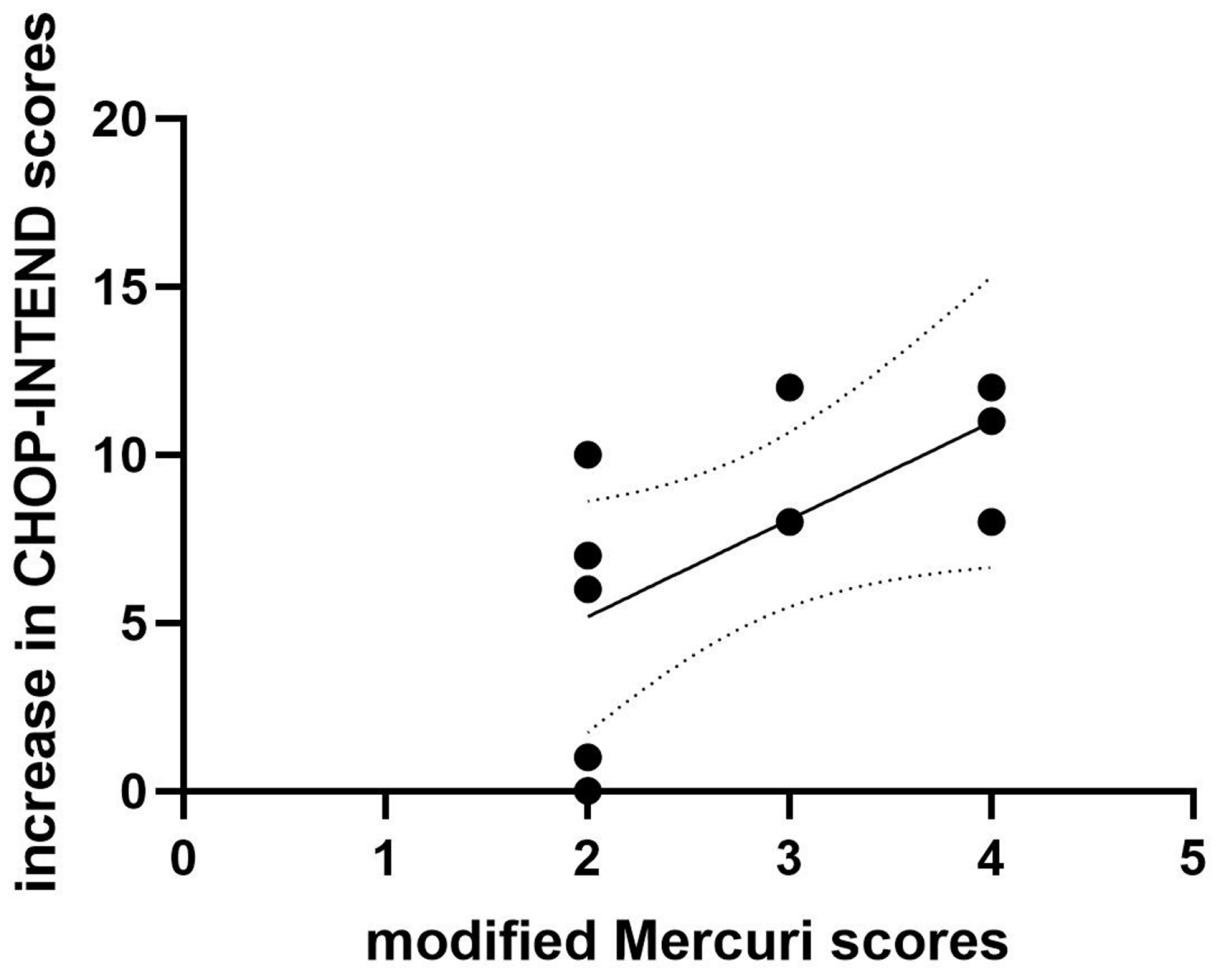
The correlation between the modified Mercuri scores at baseline and increase in CHOP-INTEND scores in rehabilitation group. There is a positive correlation between the modified Mercuri scores at baseline and increase in CHOP-INTEND scores (r = 0.702, *p* = 0.024).

The modified Mercuri grading system was applied to assess the degree of fatty infiltration in the quadriceps muscles of the patients. A correlation analysis was conducted between the modified Mercuri scores at baseline in the patients and the increase of CHOP-INTEND scores after 14 months of treatment (Figure 4). In the rehabilitation group, there is a positive correlation between the two (r = 0.702, *p* = 0.024).

**Figure 5:**
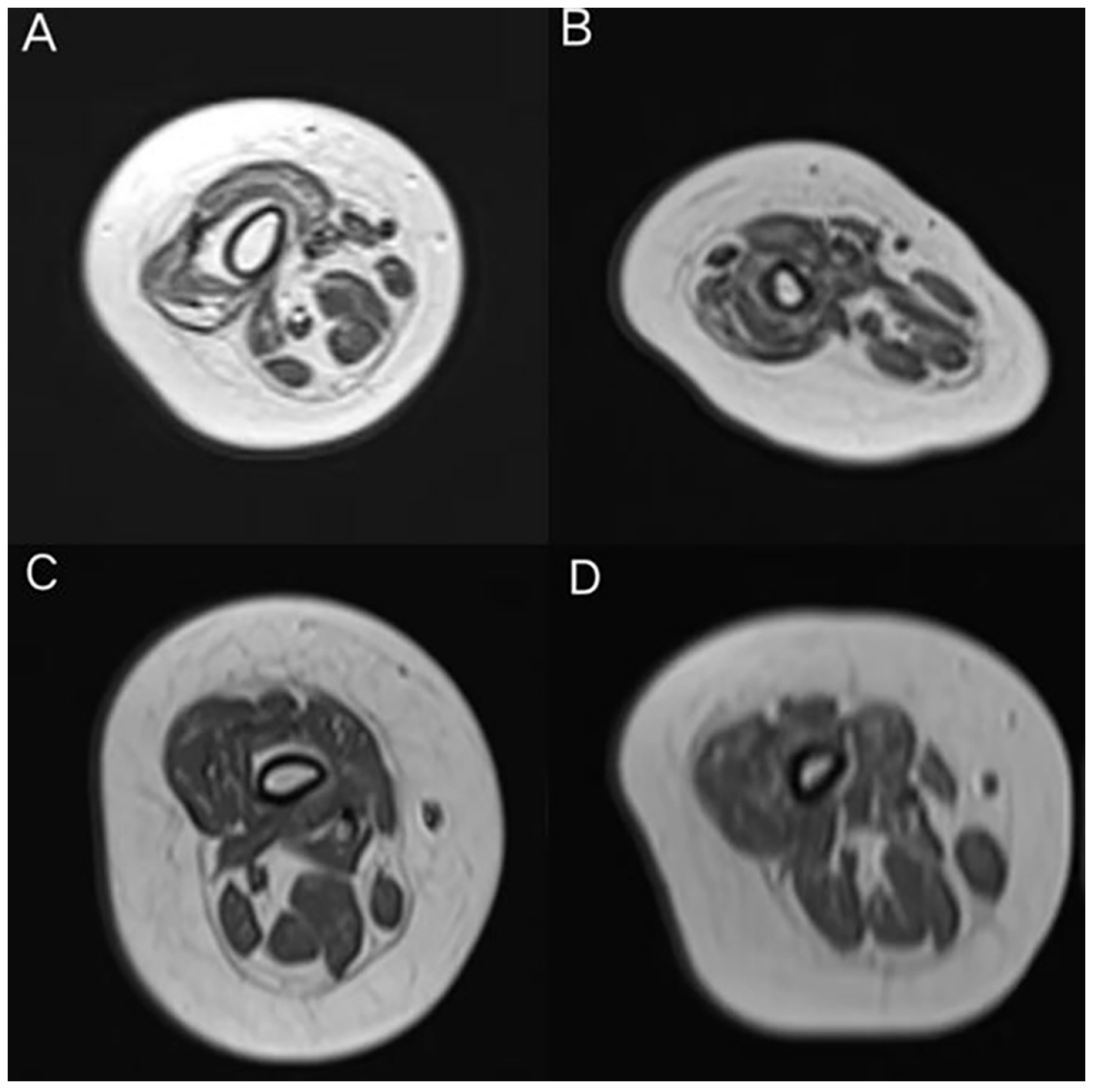
MRI images of the thigh muscles of a patient from the rehabilitation group at baseline **(A)** and at 14 months of nusinersen treatment **(B)**. At 14 months, there was a slight alleviation in the degree of fat infiltration in the quadriceps femoris (from grade 4 to grade 3). MRI images of the thigh muscles of a patient from the non-rehabilitation group at baseline **(C)** and at 14 months of nusinersen treatment **(D)**. There was no significant change in the degree of muscle fat infiltration between baseline and 14 months.

The grade of the two groups of patients were compared, and the results are shown in Table 4. There were no significant differences in the modified Mercuri scores between the two groups at baseline and at 14 months of treatment (*p* = 0.159 and *p* = 0.405, respectively). However, within the rehabilitation group, one patient showed mild alleviation in the degree of fatty infiltration in the quadriceps muscles at 14 months of treatment (from grade 4 to grade 3), while no such improvement was observed in the patients of the non-rehabilitation group (Figure 5).

**Table 4:** The modified Mercuri grade of the quadriceps muscles in the rehabilitation.

	Baseline						14 months					
	Rehabilitation therapy			Non-rehabilitation therapy			Rehabilitation therapy			Non-rehabilitation therapy		
Grade/statistics	2	3	4	2	3	4	2	3	4	2	3	4
*n* (%)	5 (50.0)	2 (20.0)	3 (30.0)	1 (14.3)	2 (28.6)	4 (57.1)	1 (25.0)	1 (25.0)	2 (50.0)	0 (0.0)	1 (25.0)	3 (75.0)
*p* value	0.159						0.405					

Therapy group vs. the non-rehabilitation therapy group.

During the study, no nusinersen-related abnormalities in electrocardiograms, liver and kidney function, or coagulation function were observed. One (4.0%) patient experienced headache after one injection, and two patients (8.0%) experienced back pain, which resolved without treatment. These symptoms were likely unrelated to nusinersen and could be attributed to the lumbar puncture procedure. No gastrointestinal reactions or allergic reactions were observed.

## 4 Discussion

Spinal muscular atrophy (SMA) is one of the most severe genetic diseases. In the natural course of SMA, this disease leads to premature death (in acute forms) or severe motor disability (in chronic forms) (27). The natural course of SMA is highly dependent on its subtype: patients with SMA type2 experience a plateau in motor milestones after 18–24 months of age, followed by a progressive loss of independent sitting ability, with an annual decline in pulmonary function of approximately 4%–6% (28). Prior to the availability of target treatment, rehabilitation therapy was provided to patients with SMA, which is effective but cannot reverse disease progression or alter the natural history of SMA. The approval of nusinersen as a SMA-targeting drug has introduced a new era of SMA treatment, and multiple studies have already demonstrated the effectiveness of nusinersen for this disease (29, 30). This study provided evidence that adding rehabilitation therapy to nusinersen improved clinical outcomes, and the levels of pNF-H and thigh skeletal muscle MRI can serve as potential biomarkers for evaluating the effectiveness of SMA treatment.

According to the latest research and data, the efficacy of Nusinersen in treating SMA is indeed closely related to the age at which patients begin treatment. Prospective cohort study conducted by Pechmann et al. (31) included a total of 61 children with Type 1 SMA, with an average age of 21.08 months. After 6 months of treatment, patients who started treatment before 7 months of age showed a greater increase in CHOP-INTEND scores compared to those who started after 7 months (14.4 and 7.0). In our study, there was a negative correlation observed between the age at medication initiation, the medication time window, and the improvement in CHOP-INTEND scores in SMA 2 patients. It is possible that as the disease progresses and functional SMN proteins degenerate, the patients lose motor function gradually This suggests that the younger the age at medication initiation and the shorter the medication time window, the better the therapeutic effect of nusinersen. This highlights the importance of early medication intervention. Therefore, it is recommended that all eligible SMA patients, especially those with early symptom onset, promptly discuss and initiate treatment with their healthcare providers.

Previously, research by Mirea et al. (13) indicated that the combination of Nusinersen with rehabilitation therapy achieves better treatment outcomes compared to the use of Nusinersen alone, especially in terms of motor function. In our study, although significant improvements were observed in motor function based on various scales among all patients with SMA type 2 treated with nusinersen, Patients who received rehabilitation therapy demonstrated greater improvements in CHOP-INTEND and RULM scores compared with those who did not receive rehabilitation therapy. The intergroup differences in scores indicated that rehabilitation therapy may have significantly improved the effectiveness of nusinersen on type 2 SMA patients, especially fine motor skills and upper limb function, although achieving milestones in motor function may be challenging within the relatively short observation period. Although the overall swallowing function in patients with SMA type 2 remained stable after treatment, individual patients showed improved swallowing function, characterized by an increased variety of foods that could be consumed. The improvement may have contributed to better nutrition levels and improved quality of life. It is important to note that both the CHOP INTEND and RULM scales have clear upper score limits (e.g., CHOP INTEND has a maximum score of 64). The higher baseline scores in the non-rehabilitation group (CHOP INTEND: 43 vs. 39) may have limited their potential for improvement. Over the first 6 months, the rehabilitation group showed an improvement (from 39 to 45), whereas the non-rehabilitation group improved from 43 to 45.7. This is consistent with the “ceiling effect” observed in previous SMA studies (32). Further analysis using covariance analysis to adjust for baseline scores revealed that the functional improvement in the rehabilitation group at 6 months remained significantly superior to that of the non-rehabilitation group (ΔCHOP INTEND: 6.0 vs. 2.7, *p* = 0.032*). This suggests that the positive effects of rehabilitation training are independent of baseline differences. The plateauing of scores in the rehabilitation group after 6 months may be related to a shortened window of neuroplasticity or the phase-specific saturation of motor function compensation mechanisms.

Regarding the research on pNF-H as a biomarker for SMA, there have been some international achievements. Darras et al. (16) found that SMA children had elevated plasma pNF-H levels through testing a group of SMA children’s plasma, and Nusinersen treatment was significantly associated with a decrease in pNF-H levels. In our study, after 14 months of treatment, the levels of pNF-H in the serum and cerebrospinal fluid of patients with SMA showed a significant decrease compared with baseline, which may reflect a reduction in motor neurons and axonal degeneration. And the change in pNF-H concentration in the cerebrospinal fluid is negatively correlated with the improvement of CHOP-INTEND scores. This indicates that pNF-H can serve as a potential biomarker for SMA. However, previous studies showed that the levels of pNF-H in the serum of patients with SMA also decreased over time without nusinersen treatment, albeit at a slower rate compared with those receiving nusinersen treatment (23). It is worth noting that the variability in results may be related to differences in the sensitivity of the ELISA kits used (33). There were no significant differences in pNF-H levels between the two groups of patients, suggesting that although rehabilitation therapy can improve muscle strength and motor function, it may not have a significant impact on reducing motor neurons. Due to ethical considerations (minimizing blood draws in infants) and the initial study design, our study did not include pNF-H measurements at 2–3 months of treatment. Future studies should track pNF-H dynamics at more frequent time points (e.g., 0, 1, 3, and 6 months). And the study included a broad age range (1–9.5 years) but did not conduct subgroup analyses. These limitations need to be further optimized in future studies.

Douglas et al. (34). measured muscle volume by MRI in 11 SMA patients, distinguishing between normal and abnormal signal intensities, and analyzed their correlation with motor function. The results indicated a strong positive correlation between motor function measurements and muscle volume of normal signal intensity, suggesting that muscle MRI can serve as a biomarker for treatment efficacy. In the rehabilitation group, there was a positive correlation between the modified Mercuri scores at baseline and the improvement in CHOP-INTEND scores after 14 months of treatment. Patients with more severe muscle atrophy at baseline showed more significant improvement in CHOP-INTEND scores after receiving nusinersen combined with rehabilitation treatment. This may be related to the ceiling effect of the CHOP-INTEND scores, and it also highlights the necessity of nusinersen combined with rehabilitation treatment in patients with severe muscle atrophy. There were no significant differences in the modified Mercuri grade of the quadriceps muscles between the two groups of patients at baseline and 14 months of treatment. This may be attributed to the small sample size, short observation period, and the difficulty in alleviating muscle atrophy in some patients, especially those with older age. One SMA patient who received rehabilitation treatment showed improvement in the degree of fatty infiltration of muscle after 14 months of treatment compared to baseline. This provides some evidence that rehabilitation treatment can alleviate muscle atrophy in SMA patients. Regular monitoring of thigh skeletal muscle MRI may serve as an observational indicator for evaluating the effectiveness of rehabilitation treatment. The high dropout rate in MRI follow-up in this study (baseline *n* = 17 vs. 14 months *n* = 8) weakened the reliability of the results. This was mainly due to the poor tolerance of pediatric subjects, leading to a high mid-examination dropout rate in whole-body MRI. We acknowledge the limitations of MRI assessment and that further research is needed in this area. Further validation is still needed with a larger and more diverse population, as well as a longer duration of observation.

These safety findings were consistent with the safety profile of nusinersen combined with rehabilitation therapy. Apart from a few cases of headache and back pain following the intrathecal injection, no other adverse reactions were observed. The headaches and back pain were more likely related to the lumbar puncture procedure than the medication itself and resolved relatively quickly with rest.

This study had several limitations. First, only patients with SMA type 2 were included, which limits the generalizability of conclusions to other SMA types. Second, the follow-up period was relatively short, and a longer duration of follow-up may allow more pronounced changes to be observed. Third, serum and cerebrospinal fluid samples were not collected at every time point, resulting in a lack of continuity in pNF-H data. These aspects could be improved in future studies.

In conclusion, our study provided evidence that rehabilitation improves the effectiveness of nusinersen on type 2 SMA patients, and the levels of pNF-H and thigh skeletal muscle MRI can serve as potential biomarkers for evaluating the effectiveness of SMA treatment.

## Data Availability

The original contributions presented in the study are included in the article/supplementary material, further inquiries can be directed to the corresponding authors.
